# Advanced liver fibrosis is associated with decreased gait speed in older patients with chronic liver disease

**DOI:** 10.1038/s41598-024-57342-1

**Published:** 2024-03-21

**Authors:** Kenichi Fudeyasu, Kai Ushio, Takuo Nomura, Toshihiro Kawae, Daisuke Iwaki, Yuki Nakashima, Akiko Nagao, Akira Hiramatsu, Eisuke Murakami, Shiro Oka, Yukio Mikami

**Affiliations:** 1 Division of Rehabilitation, Department of Clinical Practice and Support, Hiroshima University Hospital, Hiroshima, Japan; 2 Department of Rehabilitation Medicine, Hiroshima University Hospital, Hiroshima, Japan; 3 Department of Physical Therapy, Faculty of Rehabilitation, Kansai Medical University, Osaka, Japan; 4 Department of Physical Therapy, Makuhari Human Care Faculty, Tohto University, Chiba, Japan; 5 Division of Nutrition Management, Hiroshima University Hospital, Hiroshima, Japan; 6 Department of Gastroenterology, KKR Hiroshima Memorial Hospital, Hiroshima, Japan; 7 Department of Gastroenterology, Hiroshima University Hospital, Hiroshima, Japan

**Keywords:** Gait speed, Liver disease, Liver fibrosis, Muscle strength, Sarcopenia, Diseases, Gastroenterology, Health care, Medical research, Signs and symptoms

## Abstract

This study investigated whether the progression of liver fibrosis affects the prevalence of sarcopenia and incidence of decreased gait speed in older patients with chronic liver disease (CLD). Patients with CLD aged ≥ 60 years were classified into low, intermediate, and high fibrosis 4 (FIB-4) index groups according to the degree of liver fibrosis. The prevalence of sarcopenia and incidence of decreased gait speed (< 1.0 m/s) were compared among the three groups. Logistic regression analysis was performed to investigate factors affecting the risk of decreased gait speed. No significant difference was observed in the prevalence of sarcopenia among the three groups, but the incidence of decreased gait speed significantly differed (*p* = 0.029). When analyzed individually, a significant difference in decreased gait speed incidence was observed between the high and low FIB-4 index groups (*p* = 0.014). In logistic regression analysis, the progression of liver fibrosis (odds ratio: 1.32, 95% confidence interval: 1.13–1.55) and lower extremity muscle strength (LEMS) (odds ratio: 0.92, 95% confidence interval: 0.88–0.97) were significantly associated with decreased gait speed. As liver fibrosis progresses in older patients with CLD, it becomes important to focus on not only skeletal muscle mass and grip strength, but also gait speed and LEMS.

## Introduction

Worldwide, 1.5 billion people experience chronic liver disease (CLD), and the incidence of CLD and cirrhosis has increased by 13% since 2000^1^. The incidence of CLD increases and the prognosis worsens with age^2^. CLD is a condition in which various factors cause liver fibrosis, leading to liver injury and cirrhosis^3^. The financial burden of CLD increases with a decline in patient quality of life and activities of daily living (ADL) during disease progression^4–6^. In Japan, the number of older patients with CLD is increasing because of the aging population, as well as advances in treatment^7,8^.

Sarcopenia is induced by aging, nutritional status, cachexia, and various diseases^9^. In recent years, sarcopenia has become an important topic in the field of care prevention, particularly for the growing older population^10^. The European Working Group on Sarcopenia in Older People (EWGSOP) and Asian Working Group for Sarcopenia (AWGS) recommend the diagnosis and classification of the severity of sarcopenia based on skeletal muscle mass, muscle strength, and physical function. Recently, new criteria for sarcopenia have been published in the EWGSOP 2 and AWGS 2019 guidelines^11,12^.

Sarcopenia is a strong risk factor for mortality in older patients with CLD^13–15^. The prevalence of sarcopenia increases with the severity of cirrhosis, an advanced state of CLD^14^. Therefore, it is important to clarify the association between the development of liver fibrosis and sarcopenia in the process of CLD leading to cirrhosis. However, there are few reports on the association between the progression of liver fibrosis and sarcopenia in CLD. Specifically, there are no reports on the association between the progression of liver fibrosis and physical function measures such as gait speed in CLD, although skeletal muscle mass tends to decrease in patients with advanced liver fibrosis^16^ and that liver fibrosis severity is associated with lower grip strength^17^. The Japanese Society of Hepatology (JSH) has established criteria for assessing sarcopenia; however, these criteria only include skeletal muscle mass and grip strength^18,19^.

The use of criteria that focus on not only skeletal muscle mass and muscle strength but also physical function measures, such as gait speed, can provide new insights into the presence and severity of sarcopenia in older patients with CLD leading to cirrhosis and the relationship between the progression of liver fibrosis and physical function. An understanding of the relationship between the progression of liver fibrosis and gait speed in older patients with CLD would be useful in the prevention and improvement of sarcopenia and decline in physical function. Therefore, the purpose of this study was to examine the association between the progression of liver fibrosis and sarcopenia in older patients with CLD using the latest sarcopenia criteria. In addition, the association between the progression of liver fibrosis and gait speed as a physical function measure was determined.

## Methods

### Study design and patients

**Figure 1:**
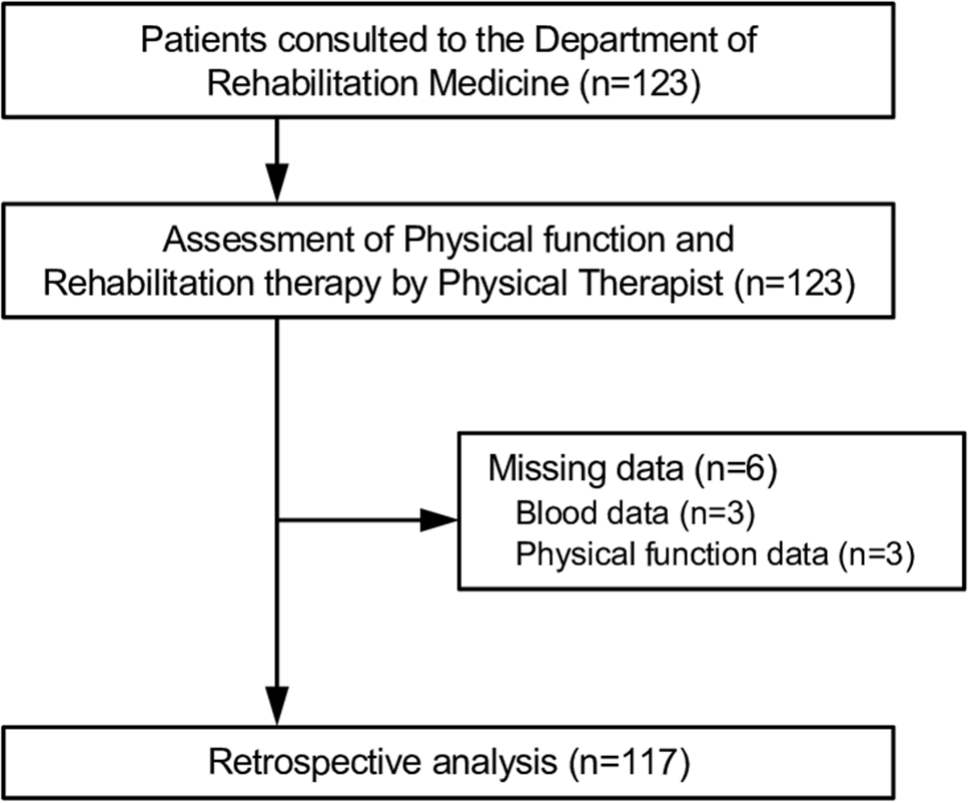
Flowchart of patient enrollment into the study.

Data for this study were collected retrospectively from medical records. Patients aged ≥ 60 years with CLD, who were admitted to the Department of Gastroenterology at Hiroshima University Hospital between April 2014 and June 2018 and referred to the Department of Rehabilitation Medicine, were included in this study. A total of 123 consecutive patients were included in this study. All patients had been hospitalized due to CLD, including hepatocellular carcinoma, diagnosed by hepatologists in our University Hospital before the assessment of physical function and rehabilitation therapy by a physical therapist. This study excluded patients with pacemaker insertions, for whom body composition assessments were not indicated, as well as those with neuromuscular diseases, excessive pain, or dementia that impeded the assessment of physical function. Three patients were excluded because of missing measurement data due to neuromuscular diseases, excessive pain, or dementia (Fig. 1). The study was conducted in accordance with Declaration of Helsinki and the Ethical Guidelines for Medical and Health Research Involving Human Subjects enacted by the Ministry of Health, Labour and Welfare of Japan (https://www.mhlw.go.jp/content/001077424.pdf), and approved by the Ethical Review Committee of Hiroshima University Hospital (Permit No. E-583-1). All patients were verbally informed that their medical records and charts might be used for research purposes. Data were obtained during routine medical care and reviewed retrospectively. The need to obtain informed consent from the study participants was waived by the Ethical Review Committee of Hiroshima University Hospital since this is a retrospective analysis. Patients who were eligible for this study had the opportunity to refuse to participate in the study by opting out.

Data on patient characteristics, blood analysis, body composition, and physical function were collected. Assessment of patient characteristics and hematological tests were performed on admission, whereas body composition and physical function were assessed on the day of initiation of physical therapy. Skeletal muscle mass and physical function were assessed by a physical therapist in the rehabilitation room.

### Clinical and laboratory assessments

Patient age, sex, height, weight, body mass index, and blood data [hemoglobin (HGB), platelets (PLT), total bilirubin (T-BIL), aspartate aminotransferase (AST), alanine aminotransferase (ALT), albumin (ALB), prothrombin time (PT), ammonia, and hemoglobin A1c] were extracted from the medical records. Diagnoses of chronic hepatitis, liver cirrhosis, encephalopathy, ascites, hepatocellular carcinoma (HCC), hypertension (HT), dyslipidemia (DL), diabetes mellitus (DM), chronic heart failure (CHF), and cardiovascular disease (CVD) were also recorded.

### Liver fibrosis assessments

The indices of liver fibrosis used in this study as a scoring system with serum markers included AST-platelet ratio index (APRI) and fibrosis 4 (FIB-4) index^20–23^.

The FIB-4 index was used as a representative biomarker to determine the pathological progression of CLD and to assess the degree of liver fibrosis. The FIB-4 index was calculated using the following formula^24–26^:

$$FIB{\text{-}}4\;index = \frac{{AST\left[ \frac{U}{L} \right] \times age \left[ {years} \right]}}{{PLT \left[ {\frac{{10^{9} }}{L}} \right] \times \sqrt {ALT \left[ \frac{U}{L} \right] } }}.$$

The FIB-4 index provides information regarding the risk of fibrosis^24^. The patients in this study were categorized into three groups based on the FIB-4 index in accordance with previous studies^27,28^: low (FIB-4 index < 2.0), intermediate (2.0 ≤ FIB-4 index ≤ 2.67), and high (FIB-4 index > 2.67) FIB-4 index groups.

In addition, to verify the validity of the FIB-4 index, we calculated the APRI, which is another indicator of liver fibrosis, using the following formula:

$$APRI = \frac{{AST\left[ \frac{U}{L} \right]/upper\;limit\;of\;normal\;AST\left[ \frac{U}{L} \right]}}{{PLT \left[ {\frac{{10^{9} }}{L}} \right]}} \times 100.$$

### Body composition assessments

The skeletal muscle mass was measured using a bioelectrical impedance assessment (BIA) device (InBody720; InBody Co., Ltd., Seoul, Korea). The sum of extremity muscle mass was calculated as Appendicular skeletal muscle mass (ASM). The skeletal muscle mass index (SMI) was calculated as ASM divided by the square of height^12,18,19^.

To measure intracellular and extracellular water content, the ratio of extracellular water to total body water (ECW/TBW) was calculated.

### Muscle strength assessments

Upper and lower extremity muscle strength were assessed. Upper extremity muscle strength was measured using grip strength based on the AWGS 2019 criteria. Grip strength was measured in 0.5 kg units using a grip strength meter (Smedley-type grip strength meter; Matsuyoshi Medical Instruments Co., Tokyo, Japan). Patients were instructed to stand with their elbows extended and their upper limbs lowered. Two measurements were obtained for each upper extremity, and the average of the highest grip strength values obtained for each side was used in the analyses.

Lower extremity muscle strength (LEMS) assessed the knee extensor force (KEF) using a belt-mounted handheld dynamometer (μTas F-1; Anima, Tokyo, Japan). Patients were instructed to sit in the end-seated position with an upright trunk and one lower leg secured to the belt, with the knee joint flexed at 90º. The maximum isometric knee extension strength was then measured. Two measurements were taken on each side, and the average of the maximum values obtained for each side was used in the analyses. To account for differences in body size, KEF was divided by the patient’s body weight or the lower extremity muscle mass^29,30^. The KEF/weight was multiplied by 100 at the time of its input, as described in a previous study^31^.

### Gait speed assessment

The participants were instructed to walk for 16 m. The first 3 m allowed them to pick up speed while the final 3 m allowed them to slow down. The time required to walk the middle 10 m was measured using a stopwatch, and the gait speed was calculated and used in the analyses. The patients were not permitted to use a walking aid and were instructed to walk normally at a comfortable pace^11,12^. The time required to walk 10 m was measured twice, and the average value was used in the analyses.

According to the latest EWGSOP 2 and AWGS 2019 diagnostic criteria for sarcopenia, the cutoff value for a comfortable gait speed is < 1.0 m/s^11,12^, and we defined decreased gait speed as a speed of < 1.0 m/s.

### Diagnosis of sarcopenia

The criteria for sarcopenia defined by AWGS 2019^12^ and JSH (second version)^18,19^ were used in this study. The AWGS 2019 diagnostic criteria for skeletal muscle mass loss and decreased muscle strength or gait speed correspond to sarcopenia. The AWGS 2019 criteria for loss of skeletal muscle mass include an SMI of < 7.0 kg/m^2^ in men and < 5.7 kg/m^2^ in women, while the criteria for muscle weakness include a grip strength of < 28 kg in men and < 18 kg in women, with a decreased gait speed of < 1.0 m/s^12^.

The JSH (second version) diagnostic criteria for loss of skeletal muscle mass and muscle weakness correspond to sarcopenia. The JSH criteria for loss of skeletal muscle mass are defined as an SMI of < 7.0 kg/m^2^ in men and < 5.7 kg/m^2^ in women, while the criteria for muscle weakness include a grip strength of < 28 kg in men and < 18 kg in women^18,19^.

### Statistical analysis

Continuous variables are presented as median (interquartile range) and categorical variables are presented as number (frequency). Among the three FIB-4 index groups, continuous variables were compared using the Kruskal–Wallis test, while categorical variables were compared using Fisher's exact test. The incidence of decreased gait speed (< 1.0 m/s) was compared among the three FIB-4 index groups using Fisher’s exact test with the Bonferroni correction. Furthermore, we reviewed prior studies to determine the percentage of gait speed divided by 0.2 m/s for each Fib4 index grouping^32,33^. The association between the FIB-4 index and gait speed was investigated using Spearman’s correlation coefficient for each FIB-4 index grouping. Factors associated with decreased gait speed (< 1.0 m/s) were analyzed by logistic regression analysis using the forced entry method. The explanatory variables were age, sex, presence of skeletal muscle mass loss, FIB-4 index, presence of HCC, and KEF/weight. The patients with CLD were further subclassified into five categories according to etiology: hepatitis B virus (HBV), hepatitis C virus (HCV), alcoholic liver injury (Alc), nonalcoholic fatty liver disease (NAFLD), and others. Multiple comparisons were made using the Steel–Dwass test for continuous variables and Fisher’s exact test for nominal variables.

JMP version 17 statistical software (SAS Institute Inc., Cary, NC, USA) was used to conduct the statistical analyses in this study. All hypothesis tests were two-tailed. Statistical significance was set at *p* < 0.05, with *p* < 0.016 only for analyses with the Bonferroni correction.

## Results

### Patient characteristics

Age, sex, and BMI did not significantly differ between the three FIB-4 index groups. Similarly, the three groups showed no significant difference in the prevalence of comorbidities, such as HT, DL, DM, CHF, and CVD. The FIB-4 index (*p* < 0.001), APRI (*p* < 0.001), incidence of liver cirrhosis (*p* < 0.001), and liver reserve capacity (*p* < 0.001) were significantly different among the three FIB-4 index groups. There were significant differences in HGB (*p* = 0.042), PLT (*p* < 0.001), T-BIL (*p* < 0.001), AST (*p* < 0.001), ALB (*p* = 0.013), and PT (*p* < 0.001) among the three groups (Table 1).

**Table 1:** Characteristics of patients in the three FIB-4 index groups.

	All subjects (n = 117)	Low FIB-4 index (n = 36)	Intermediate FIB-4 index (n = 18)	High FIB-4 index (n = 63)	*p* value
Age (years)	70 (66–74)	68 (64–73)	70 (65–72)	70 (67–76)	0.145
Sex (male/female)	70 (60)/47 (40)	23 (64)/13 (36)	9 (50)/9 (50)	38 (60)/25 (40)	0.659
BMI (kg/m^2^)	24.6 (22.0–28.4)	24.8 (23.3–28.2)	27.1 (22.9–30.8)	23.8 (21.5–28.0)	0.063
≥ 25 (yes/no)	54 (46)/63(54)	16 (44)/20 (56)	12 (67)/6 (33)	26 (41)/37 (59)	0.156
Etiology (HBV/HCV/Alc/NAFLD/other)	12/49/14/38/4 (10/42/ 12/ 32/ 4)	5/15/2/12/2 (14/41/6/33/6)	3/7/1/7/0 (16/39/6/39/0)	4/27/11/19/2 (6/43/18/30/3)	0.554
FIB-4 index	2.77 (1.86–4.50)	1.49 (1.13–1.80)	2.43 (2.23–2.62)	4.25 (3.23–6.96)	< 0.001**
APRI	0.78 (0.45–1.25)	0.37 (0.26–0.52)	0.63 (0.44–1.07)	1.14 (0.81–1.77)	< 0.001**
LC (yes/no)	38 (32)/79 (68)	2 (6)/34 (94)	3 (17)/15 (83)	33 (52)/30 (48)	< 0.001**
CH/CP A/CP B/CP C	79/25/11/2 (68/21/9/2)	34/1/1/0 (94/3/3/0)	15/3/0/0 (83/17/0/0)	30/21/10/2 (48/33/16/3)	< 0.001**
Encephalopathy (yes/no)	1 (1)/116 (99)	1 (3)/35 (97)	0 (0)/18 (100)	0 (0)/63 (100)	0.462
Ascites (yes/no)	7 (6)/110 (94)	1 (3)/35 (97)	0 (0)/18 (100)	6 (10)/57 (90)	0.351
HCC (yes/no)	44 (38)/73 (62)	10 (28)/26 (72)	7 (39)/11 (61)	27 (43)/36 (57)	0.332
Complications					
HT (yes/no)	70 (60)/47 (40)	22 (61)/14 (39)	14 (78)/4 (22)	34 (54)/29 (46)	0.193
DL (yes/no)	26 (22)/91 (78)	10 (28)/26 (72)	5 (28)/13 (72)	11 (17)/52 (83)	0.382
DM (yes/no)	97 (83)/20 (17)	32 (89)/4 (11)	15 (83)/3 (17)	50 (79)/13 (21)	0.522
CHF (yes/no)	1 (1)/116 (99)	0 (0)/36 (100)	0 (0)/18 (100)	1 (2)/62 (98)	1.000
CVD (yes/no)	10 (9)/107 (91)	2 (6)/34 (94)	0 (0)/18 (100)	8 (13)/55 (87)	0.248
Blood date					
HGB (g/dL)	13.5 (12.4–14.8)	13.9 (13.2–14.9)	13.6 (13.2–15.0)	13.1 (11.7–14.4)	0.042*
PLT (10^4^/μL)	15.9 (10.7–19.9)	21.1 (17.5–27.1)	17.8 (15.6–21.9)	11.1 (7.3–15.1)	< 0.001**
T-BIL (mg/dL)	0.9 (0.7–1.1)	0.7 (0.5–1.0)	0.8 (0.7–0.9)	1.0 (0.7–1.4)	< 0.001**
AST (U/L)	30 (22–48)	22 (19–30)	35 (22–56)	34 (24–50)	< 0.001**
ALT (U/L)	25 (18–47)	27 (20–46)	27 (17–76)	24 (18–46)	0.744
ALB (g/dL)	4.2 (3.8–4.5)	4.4 (3.9–4.5)	4.3 (4.1–4.6)	4.1 (3.5–4.4)	0.013*
PT (%)	89 (77–97)	96 (87–106)	90 (85–95)	80 (68–91)	< 0.001**
NH_3_ (μmol/L)	36 (28–49)	34 (28–43)	34 (28–42)	38 (29–55)	0.279
HbA1c (%)	7.0 (6.2–7.8)	7.1 (6.5–8.1)	7.2 (6.7–7.8)	6.6 (6.1–7.5)	0.150

Continuous variables are presented as median (interquartile range). Categorical variables are presented as number (percentage).

FIB-4, Fibrosis-4; BMI, Body mass index; HBV, Hepatitis B virus; HCV, Hepatitis C virus; Alc, Alcoholic liver injury; NAFLD, Non-alcoholic fatty liver disease; APRI, Aspartate aminotransferase to platelet ratio index; LC, Liver cirrhosis; CH, Chronic hepatitis; CP, Child Pugh classification; HCC, Hepatocellular carcinoma; HT, Hypertension; DL, Dyslipidemia; DM, Diabetes mellitus; CHF, Chronic heart failure; CVD, Cardiovascular disease; HGB, Hemoglobin; PLT, Platelet; T-BIL, Total bilirubin; AST, Aspartate aminotransferase; ALT, Alanine aminotransferase; ALB, Albumin; PT, Prothrombin time; NH_3_, Ammonia; HbA1c, Hemoglobin A1c.

*p < 0.05; **p < 0.01.

### Body composition, physical function, and sarcopenia

**Figure 2:**
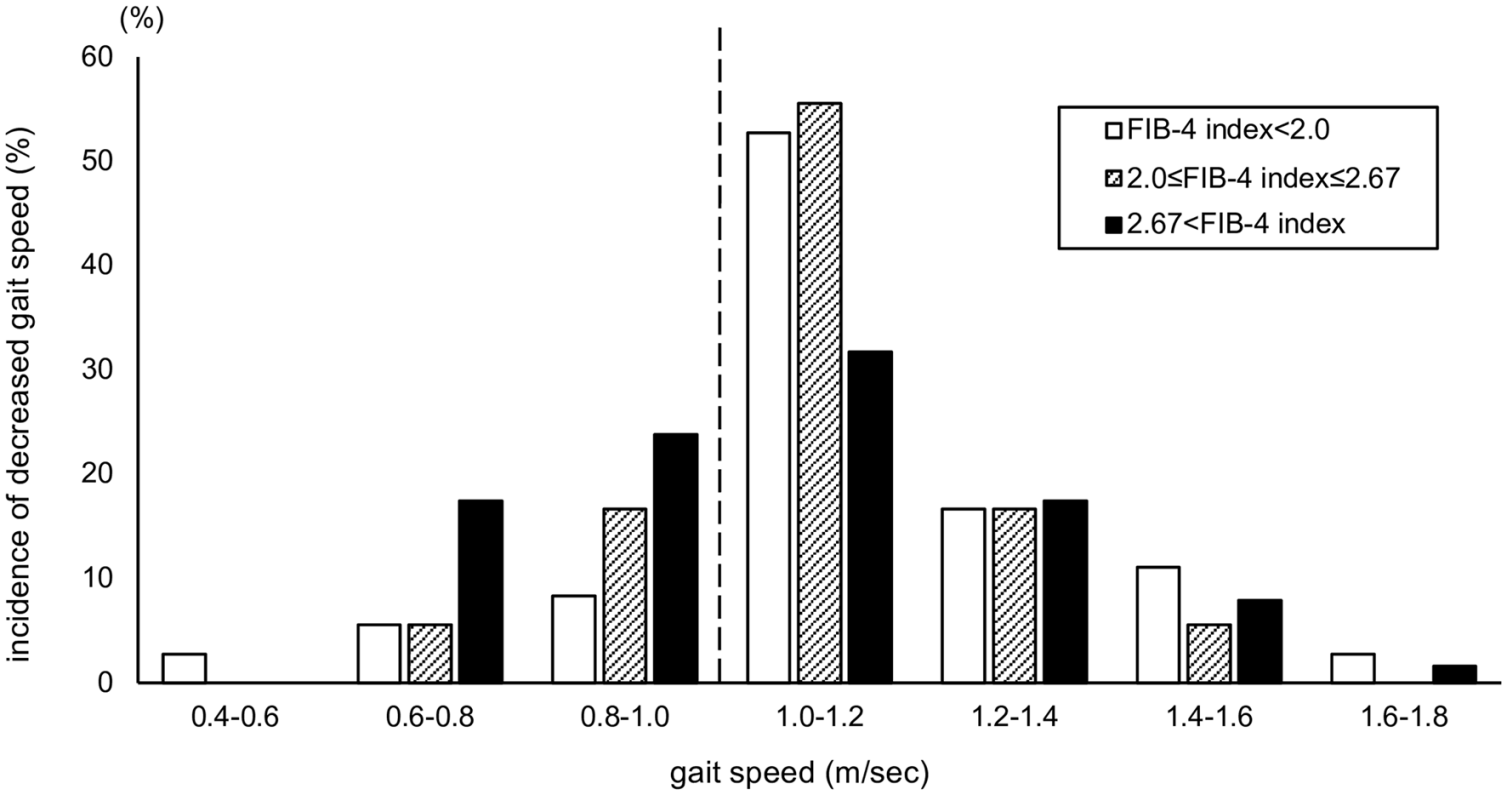
Incidence of decreased gait speed in older patients with chronic liver disease, classified according to the fibrosis 4 (FIB-4) index. The incidence of decreased gait speed in older patients with chronic liver disease classified using the FIB-4 index was 17% in the low FIB-4 index group (FIB-4 index < 2.0), 22% in the medium FIB-4 index group (2.0 < FIB-4 index < 2.67), and 41% in the high FIB-4 index group (> 2.67). The decreased gait speed was significantly higher in the high FIB-4 index group compared to the low FIB-4 index group (*p* = 0.014). In total, 5.56% and 17.46% patients in the low and high FIB-4 index groups, respectively, show a gait speed of 0.6–0.8 m/s, 8.33% and 23.81% patients in the low and high FIB-4 index groups, respectively, show a gait speed of 0.8–1.0 m/s, and 52.78% and 31.75% patients in the low and high FIB-4 index groups, respectively, show a gait speed of 1.0–1.2 m/s.

**Figure 3:**
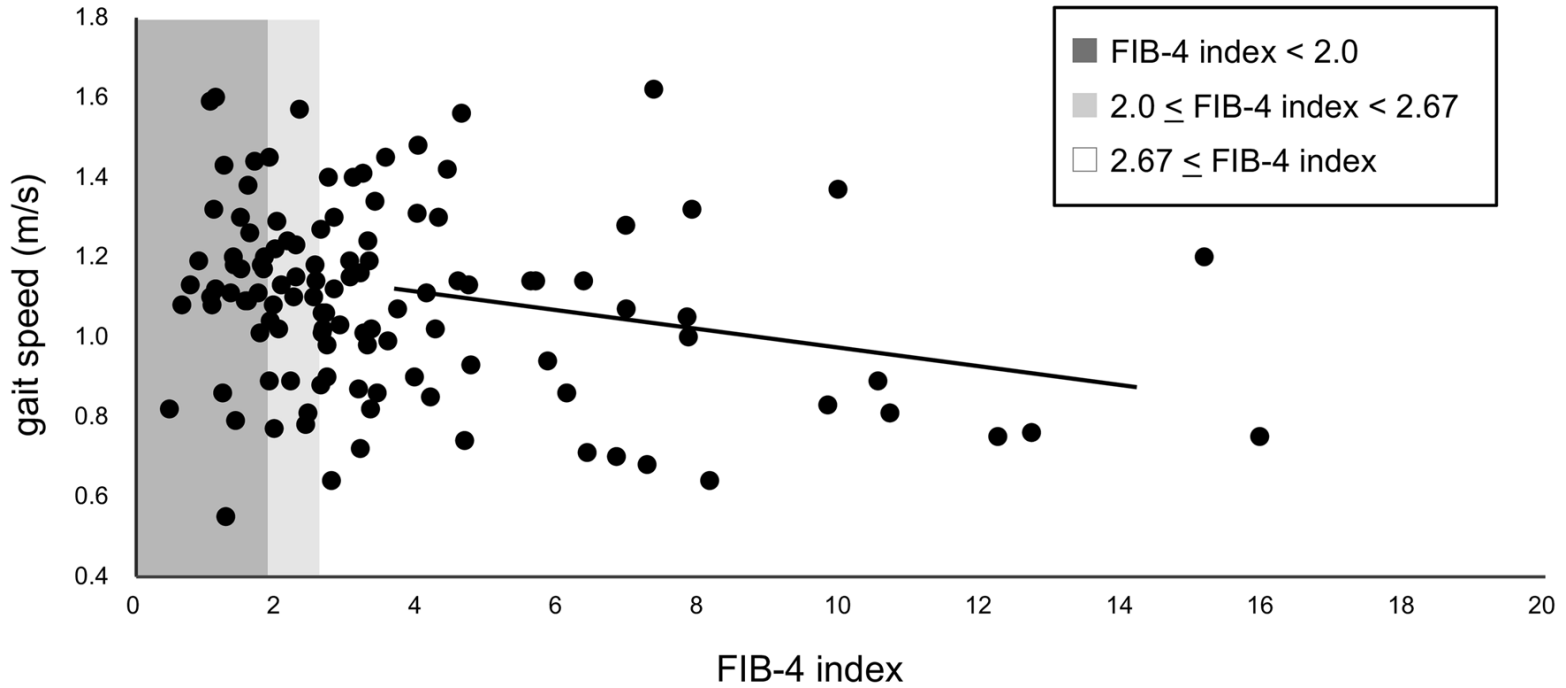
Relationship between the fibrosis 4 (FIB-4) index and gait speed. The relationship between the FIB-4 index and gait speed in older patients with chronic liver disease is shown. There is no significant correlation between gait speed and the FIB-4 index in the low FIB-4 (FIB-4 index < 2.0) and intermediate FIB-4 (2.0 ≤ FIB-4 index ≤ 2.67) index groups (ρ = 0.01; *p* = 0.939, ρ =  − 0.16; *p* = 0.514). In the high FIB-4 index group (FIB-4 index > 2.67), there is a significant correlation between gait speed and the FIB-4 index. (ρ = − 0.24; *p* = 0.047).

The prevalence of sarcopenia, determined using the AWGS 2019 criteria, was 17% among older patients with CLD in this study. The prevalence of sarcopenia according to the JSH (second version) and AWGS 2019 criteria was not significantly different among the three groups. However, there was a significant difference in the incidence of decreased gait speed among the three groups (Table 2). In multivariate analysis, the high FIB-4 index group showed a significantly higher incidence of decreased gait speed (41%) than did the low FIB-4 index group (17%; *p* = 0.014; Fig. 2). Figure 1 shows the incidence of decreased gait speed in older patients with chronic liver disease, classified according to the FIB-4 index. In total, 5.56% and 17.46% patients in the low and high FIB-4 index groups, respectively, showed a gait speed of 0.6–0.8 m/s, 8.33% and 23.81% patients in the low and high FIB-4 index groups, respectively, showed a gait speed of 0.8–1.0 m/s, and 52.78% and 31.75% patients in the low and high FIB-4 index groups, respectively, showed a gait speed of 1.0–1.2 m/s (Fig. 2). The high FIB-4 index group demonstrated a significant negative correlation between the FIB-4 index and gait speed (ρ =  − 0.24; *p* = 0.047; Fig. 3). In contrast, no significant correlation was observed in the low (ρ = 0.01; *p* = 0.939) and intermediate (ρ =  − 0.16; *p* = 0.514) FIB-4 index groups.

**Table 2:** Body composition, muscle strength, physical function, and prevalence of sarcopenia in the three FIB-4 index groups.

	All subjects (n = 117)	Low FIB-4 index (n = 36)	Intermediate FIB-4 index (n = 18)	High FIB-4 index (n = 63)	*p* value
ASM (kg)	17.79 (14.54–20.67)	18.49 (15.95–21.13)	15.59 (13.75–19.33)	17.79 (14.52–20.87)	0.252
SMI (kg/m^2^)	6.83 (6.32–7.48)	7.04 (6.52–7.48)	6.59 (5.95–7.51)	6.71 (6.17–7.47)	0.361
Male < 7.0, Female < 5.4 (yes/no)	35 (30)/82 (70)	11 (31)/25 (69)	3 (17)/15 (83)	21 (33)/42 (67)	0.417
ECW/TBW	0.391 (0.385–0.396)	0.389 (0.383–0.395)	0.391 (0.385–0.398)	0.392 (0.387–0.397)	0.234
KEF (kgf)	32.1 (25.3–39.5)	36.5 (27.0–41.4)	31.9 (25.2–44.6)	30.5 (24.7–.35.8)	0.276
KEF/weight (kgf/kg)	0.54 (0.43–0.62)	0.55 (0.44–0.63)	0.48 (0.43–0.64)	0.54 (0.41–0.60)	0.774
Grip strength (kg)	24.8 (19.5–32.1)	25.6 (20.8–34.2)	22.6 (19.4–32.5)	24.3 (18.5–30.8)	0.351
Male < 28, Female < 18 (yes/no)	41 (35)/76 (65)	11 (31)/25 (69)	4 (22)/14 (78)	26 (41)/37 (59)	0.296
Gait speed (m/s)	1.10 (0.90–1.24)	1.15 (1.08–1.28)	1.10 (0.98–1.19)	1.05 (0.86–1.24)	0.134
< 1.0 m/s (yes/no)	36 (31)/81 (69)	6 (17)/30 (83)	4 (22)/14 (78)	26 (41)/37 (59)	0.029*
0.4–0.6 m/s (%)	n = 1	2.78	0	0	
0.6–0.8 m/s (%)	n = 14	5.56	5.56	17.46	
0.8–1.0 m/s (%)	n = 21	8.33	16.67	23.81	
1.0–1.2 m/s (%)	n = 49	52.78	55.56	31.75	
1.2–1.4 m/s (%)	n = 20	16.67	16.67	17.46	
1.4–1.6 m/s (%)	n = 10	11.11	5.56	7.94	
1.6–1.8 m/s (%)	n = 2	2.78	0	1.59	
Sarcopenia AWGS2019(yes/no)	20 (17)/97 (83)	7 (19)/29 (81)	2 (11)/16 (89)	11 (17)/52 (83)	0.796
Sarcopenia JSH2nd (yes/no)	18 (15)/99 (85)	6 (17)/30 (83)	2 (11)/16 (89)	10 (16)/53 (84)	0.941

Continuous variables are presented as median (interquartile range). Categorical variables are presented as number (percentage).

FIB-4, Fibrosis 4; ASM, Appendicular skeletal muscle mass; SMI, Skeletal muscle mass index; ECW, Extracellular water; TBW, Total body water; ECW/TBW, Ratio of extracellular water to total body water; KEF, Knee extension force; AWGS, Asian Working Group for Sarcopenia; JSH, Japan Society of Hepatology.

*p < 0.05.

### Multivariate analysis of decreased gait speed

The FIB-4 index (odds ratio [OR]: 1.32, 95% CI 1.13–1.55) and KEF/weight (OR 0.92, 95% CI 0.88–0.97) were significantly associated with decreased gait speed (< 1.0 m/s), independent of age, sex, presence of HCC, and skeletal muscle mass (Table 3).

**Table 3:** Logistic regression analysis of factors for gait speed < 1.0 m/s.

Objective variable	Multivariate	OR (95% CI)	*p* value
Gait speed < 1.0 m/s	Age (years)	1.03 (0.95–1.13)	0.432
	Sex (male/female)	0.63 (0.21–1.80)	0.432
	SMI; male < 7.0, female < 5.4 (yes/no)	1.77 (0.58–5.95)	0.325
	FIB-4 index	1.29 (1.11–1.53)	< 0.001*
	HCC (yes/no)	1.04 (0.37–2.91)	0.944
	KEF/weight (kgf/kg) × 100	0.92 (0.88–0.96)	< 0.001*

OR, Odds ratio; CI, Confidence interval; SMI, Skeletal muscle mass index; FIB-4, Fibrosis-4; HCC, Hepatocellular carcinoma; KEF, Knee extension force.

*p < 0.01.

### Body composition, physical function, and sarcopenia by etiology

Supplementary Table S1 shows results of the multiple comparisons test for the characteristics of patients with CLD, categorized according to etiology. A significant difference was observed in the proportion of etiological factors between men and women (*p* < 0.05; HBV, 77% and 23%; HCV, 59% and 41%; Alc, 100% and 0%; NAFLD, 37% and 63%, respectively). Patients with NAFLD also had a significantly higher BMI than those with HCV (*p* < 0.05) and Alc (*p* < 0.05). In contrast, there were no significant differences in decreased gait speed and prevalence of sarcopenia when classified by etiology.

## Discussion

In the present study, we investigated whether the progression of liver fibrosis affects sarcopenia and gait speed as a physical function measure in older patients with CLD. The results showed that the prevalence of sarcopenia did not change with the progression of liver fibrosis, although the incidence of decreased gait speed (< 1.0 m/s) was significantly changed. Multivariate analysis revealed that the risk of decreased gait speed was associated with liver fibrosis and LEMS, regardless of age, sex, presence of hepatocellular carcinoma, and skeletal muscle mass.

The prevalence of sarcopenia was 17% among the older patients with CLD in the present study according to the latest AWGS 2019 diagnostic criteria, which include physical function as well as skeletal muscle mass, and there was no difference in the prevalence of sarcopenia according to the degree of progression of liver fibrosis. Similarly, using the JSH (second version) diagnostic criteria, we found no significant difference in the prevalence of sarcopenia according to the degree of progression of liver fibrosis. In a meta-analysis of patients with cirrhosis, the prevalence of sarcopenia as defined by skeletal muscle mass only was 37.5%^14^. In a study of patients with CLD, including hepatitis and cirrhosis, the prevalence of sarcopenia as defined by skeletal muscle mass only was 25%^34^. In the present study, which also included hepatitis and cirrhosis, approximately 30% of patients were diagnosed with sarcopenia when assessed by skeletal muscle mass only, using the AWGS2019 and JSH (second version) criteria. This result was consistent with the findings of a previous study^34^. Most sarcopenia studies involving CLD assessed the presence of sarcopenia on the sole basis of skeletal muscle mass^14,34,35^. However, if the criteria include physical function measures, particularly gait speed, the percentage of patients diagnosed with sarcopenia is likely to be lower, as observed in the present study. In addition, there are various methods and sites for evaluating skeletal muscle mass, and a unified method for diagnosis is needed in the future. In the present study, skeletal muscle mass was evaluated using BIA, and there is a possibility that water content affected the results. In liver disease, fluid retention becomes a concern as the disease progresses^36–38^.

In the present study, there was a difference in the incidence of decreased gait speed depending on the progression of liver fibrosis in older patients with CLD. Although it has been reported that the development of liver fibrosis affects skeletal muscle loss^39^, to our knowledge, this is the first study to show that the degree of liver fibrosis is associated with decreased gait speed. Gait speed is an important diagnostic criterion for sarcopenia in the EWGSOP and AWGS guidelines^11,12^. The overall mean gait speed in the present study was 1.10 m/s, and 31% of patients had a speed of < 1.0 m/s. In a cohort study of 2,000 community-dwelling older people in Asia, the average gait speed of all subjects was 1.14 ± 0.25 m/s. The values of the present study are equivalent to or slightly smaller than those in the previous large-scale study^40^. Nishikawa et al. reported a gait speed of < 1.0 m/s in approximately 10% of Japanese patients with CLD^32^; this was a smaller percentage than that observed in the present study. We believe that the study by Nishikawa et al.^32^ was influenced by the fact that it included a greater number of younger patients with an average age of 66 years (22–94 years) than did the present study. A decreased gait speed occurs with aging, particularly after the age of 60 years^41^. Decreased gait speed plays a crucial role in determining the prognosis of older individuals^42,43^, and it is also a significant factor linked to mortality in patients with liver cirrhosis^44^. Each increase of 0.1 m/s in gait speed is associated with a 12% reduction in the risk of death^43^. Gait speed is also a predictor of ADL such as bathing and dressing^43,45^. If the disease progresses, CLD causes greater physical dysfunction and ADL limitations than does osteoarthritis, diabetes mellitus, or cardiac disease^46^. Therefore, it is very important to focus on not only skeletal muscle mass but also gait speed in terms of prognosis and ADL in patients with CLD.

The risk of decreased gait speed in older patients with CLD was found to be associated with the decline in LEMS and the development of liver fibrosis, independent of age, sex, the presence of HCC, and skeletal muscle mass. In the three FIB-4 index groups, differences were observed in blood data such as HGB and ALB. Gait speed has been influenced by LEMS in the general older population^47^, and it is likely to be similarly relevant in older patients with CLD. In terms of gait speed and LEMS, there is no correlation between muscle strength and gait speed in robust older people, although there is a correlation in frail older people^48^. In general, worsening liver fibrosis leads to blood cell loss and decreased protein synthesis. In patients with frailty and sarcopenia, HGB and ALB levels are reportedly associated with decreased gait speed^49–51^. Therefore, the decrease in HGB and ALB due to the development of liver fibrosis may have affected decreased gait speed in the present study. In addition, skeletal muscle mass was not related to decreased gait speed in the present study. Physical function, including gait speed, correlates with muscle strength rather than skeletal muscle mass in patients with cirrhosis^52^. Additionally, skeletal muscle mass measured using BIA and gait speed are not correlated in patients with severe liver disease^53^. These conclusions support our finding of no relationship between decreased gait speed and skeletal muscle mass in the present study. We believe that not only skeletal muscle mass and grip strength but also LEMS should be used to evaluate gait speed in older patients with CLD. The effect of interventions on gait speed in patients with CLD is unclear. As such. it is important to pay attention to exercise physiology, physical activity, and diet in future intervention studies^54^.

There are some limitations in the present study. First, this was a single-center, cross-sectional study. Second, the sample size was small and susceptible to bias; thus, the findings may not be generalizable to other populations. Third, patients with CLD of various etiologies were included. The differences in etiologies might have affected the incidence and mechanisms of physical dysfunction. Fourth, the age of the patients, progression of liver disease, and rate of cirrhosis differed from those in previous studies. Therefore, future studies should longitudinally examine the effect of the progression of liver fibrosis on physical function in patients with CLD according to the etiology of CLD, with patients ranging from those with a recent diagnosis of hepatitis to those with progression to cirrhosis.

## Conclusions

In older patients with CLD, there was no difference in the prevalence of sarcopenia, according to criteria that included gait speed, as liver fibrosis progressed, although there was a significant difference in the incidence of decreased gait speed. The risk of decreased gait speed was associated with a decline in LEMS and progression of liver fibrosis, independent of skeletal muscle mass. It is important to consider not only skeletal muscle mass but also gait speed and LEMS, which is not a diagnostic criterion for sarcopenia.

### Supplementary Information

Supplementary Information.

## Data Availability

As the data contain potentially sensitive information, the data are not publicly available. Qualified researchers may apply for access to a minimal dataset by contacting Kenichi Fudeyasu, first author, fudeyasu@hiroshima-u.ac.jp or Kai Ushio, corresponding author, ushiosista@hiroshima-u.ac.jp.
