# Supplementary material for: Advanced liver fibrosis is associated with decreased gait speed in older patients with chronic liver disease
Source: Sci Rep. 2024 Mar 21;14:6809. doi: 10.1038/s41598-024-57342-1 (PMC10957869; doi:10.1038/s41598-024-57342-1)
Supplement: Supplementary file 1 — Supplementary Information. [file 41598_2024_57342_MOESM1_ESM.docx]

**Supplementary Table S1 Characteristics of patients with chronic liver disease by etiology**

|  | HBV | (N = 13) | HCV | (N = 51) | Alc | (N = 15) | NAFLD | (N = 32) | Others | (N = 6) |
| --- | --- | --- | --- | --- | --- | --- | --- | --- | --- | --- |
| **Age (years)** | 70 | (65–73) | 71 | (66–77) | 67 | (65–72) | 69 | (66–75) | 69 | (64–71) |
| **Sex (male/female)** | ^＊^10 (77)/3 (23) | | 30 (59)/21 (41) | | 15 (100)/0 (0) | | 12 (37)/20 (63) | | 3 (50)/3 (50) | |
| **BMI (kg/m^2^)** | 23.5 | (22.7–26.6) | 24.6^‡^ | (21.3–28.4) | 22.6^§^ | (20.6–27.4) | 27.7^‡§^ | (23.6–30.8) | 24.6 | (21.4–26.4) |
| **FIB-4 index** | 2.44 | (1.13–3.00) | 3.04 | (1.95–4.62) | 3.57 | (2.71–6.11) | 2.60 | (1.60–3.39) | 4.78 | (1.72–8.55) |
| **SMI (kg/m^2^)** | 7.449 | (6.336–7.593) | 6.593 | (6.058–7.336) | 7.322 | (6.707–7.851) | 6.822 | (6.436–7.461) | 6.847 | (5.872–7.247) |
| **KEF/weight (kgf/kg)** | 0.56 | (0.48–0.65) | 0.53 | (0.42–0.63) | 0.56 | (0.49–0.62) | 0.49 | (0.37–0.60) | 0.46 | (0.40–0.52) |
| **Grip strength (kg)** | 32.5^†^ | (24.4–37.6) | 25.0^†^ | (19.3–30.1) | 28.3 | (22.5–35.0) | 22.1 | (17.3–34.7) | 23.1 | (16.9–25.5) |
| **Gait speed (m/s)** | 1.10 | (0.92–1.21) | 1.11 | (0.94–1.22) | 1.07 | (0.98–1.31) | 1.13 | (0.85–1.24) | 1.04 | (0.83–1.30) |
| **Sarcopenia AWGS 2019** | 1 (8) | | 11 (22) | | 4 (27) | | 4 (13) | | 0 (0) | |
| **Sarcopenia JHS2nd** | 1 (8) | | 10 (20) | | 2 (13) | | 1 (3) | | 0 (0) | |

Continuous variables are presented as median (interquartile range). Categorical variables are presented as number (percentage).

＊Among the five groups, *P* <0.05, †HBV vs. HCV, *P* <0.05, ‡HCV vs. NAFLD, *P* <0.05. §ASH vs. NAFLD, *P* <0.05

HBV, hepatitis B virus; HCV, hepatitis C virus; Alc, alcoholic liver injury; NAFLD, non-alcoholic fatty liver disease; BMI, body mass index; SMI, skeletal muscle mass index; KEF, knee extension force; AWGS, Asian Working Group for Sarcopenia; JHS, the Japan Society of Hepatology
